# SOX2 expression in the pathogenesis of premalignant lesions of the uterine cervix: its histo-topographical distribution distinguishes between low- and high-grade CIN

**DOI:** 10.1007/s00418-022-02145-6

**Published:** 2022-08-09

**Authors:** Jobran M. Moshi, Monique Ummelen, Jos L. V. Broers, Frank Smedts, Koen K. Van de Vijver, Jack P. M. Cleutjens, Rogier J. N. T. M. Litjens, Frans C. S. Ramaekers, Anton H. N. Hopman

**Affiliations:** 1 Department of Molecular Cell Biology, GROW-School for Oncology and Reproduction, Maastricht University Medical Center, Maastricht, The Netherlands; 2 Department of Medical Laboratory Technology, Faculty of Applied Medical Sciences, Jazan University, Jazan, Kingdom of Saudi Arabia; 3 Department of Pathology, Cork University Hospital, Cork, Ireland; 4 Department of Pathology, CARIM-Cardiovascular Research Institute Maastricht, Maastricht University Medical Center, Maastricht, the Netherlands; 5 Department of Pathology, Ghent University Hospital, Cancer Research Institute Ghent (CRIG), Ghent, Belgium; 6 Department of Gynecology, Laurentius Hospital Roermond, Roermond, The Netherlands

**Keywords:** Cervical preneoplasia, CIN, Squamous intraepithelial lesions, SOX2 distribution, Genetic aberrations, HPV infection

## Abstract

SOX2 expression in high-grade cervical intraepithelial neoplasia (CIN3) and cervical squamous cell carcinoma is increased compared to that in the normal cervical epithelium. However, data on the expression and histological distribution of SOX2 in squamous epithelium during progression of CIN are largely lacking. We studied SOX2 expression throughout the epithelium in 53 cases of CIN1, 2, and 3. In general, SOX2 expression increased and expanded from basal/parabasal to the intermediate/superficial compartment during early stages of progression of CIN. An unexpected, specific expression pattern was found in areas classified as CIN2 and CIN3. This pattern was characterized by the absence or low expression of SOX2 in the basal/parabasal compartment and variable levels in the intermediate and superficial compartments. It was significantly associated with CIN3 (*p* = 0.009), not found in CIN1 and only seen in part of the CIN2 lesions. When the different patterns were correlated with the genetic make-up and presence of HPV, the CIN3-related pattern contained HPV-positive cells in the basal/parabasal cell compartment that were disomic. This is in contrast to the areas exhibiting the CIN1 and CIN2 related patterns, which frequently exhibited aneusomic cells. Based on their SOX2 localisation pattern, CIN1 and CIN2 could be delineated from CIN3. These data shed new light on the pathogenesis and dynamics of progression in premalignant cervical lesions, as well as on the target cells in the epithelium for HPV infection.

**Supplementary Information:**

The online version contains supplementary material available at 10.1007/s00418-022-02145-6.

## Introduction

The SOX2 transcription factor is involved in the development and differentiation of cells and tissues. It is, for example, highly expressed in pluripotent cells of the inner cell mass of the developing embryo, but SOX2 expression is also reported to be correlated with carcinogenesis, chemoresistance and maintenance of the stem cell-like phenotype in cancer cells (Liu et al. 2013; Weina and Utikal 2014; Herbert 2018; Chaudhary et al. 2019; Novak et al. 2020; Mamun et al. 2020).

Increased expression of SOX2 has been demonstrated in a range of epithelial and nonepithelial malignancies, often correlating with adverse prognostic factors, poor patient outcomes and resistance to therapies. SOX2 is involved in the tumorigenesis of squamous cell carcinoma of skin, vulvar carcinoma, gastric cancer, glioblastoma, colorectal cancer, lung cancer, oral squamous cell carcinomas and breast cancer, among others (Prince et al. 2007; Chen et al. 2008; Laga et al. 2010; Du et al. 2011; Hütz et al. 2014; Weina and Utikal 2014; Herbert 2018; Chaudhary et al. 2019). In addition, SOX2 is highly expressed in premalignant lesions, such as squamous dysplasia and carcinoma in situ in the lung (Yuan et al. 2010; Karachaliou et al. 2013; Ying et al. 2016).

SOX2-positive cells derived from cervical cancers show cancer stem cell characteristics (Ji and Zheng 2010; Stewart and Crook 2016, 2018; Herbert 2018). Furthermore, the protein can promote the proliferation, clonogenicity and tumorigenicity of cervical cancer cells in vitro (Chang et al. 2015). These findings demonstrate that SOX2 plays a role not only in the initial stages of carcinogenesis, but also in the critical steps from preneoplasia to invasive carcinoma (Stewart and Crook 2016; Herbert 2018).

SOX2 expression in high-grade cervical intraepithelial neoplasia (CIN3) and cervical squamous cell carcinomas has been reported to be higher than in the normal cervical epithelium. SOX2 expression is limited to the basal/parabasal compartment in the squamous epithelium of normal uterine cervix and is not found in immature squamous epithelium, normal endocervical columnar cells or reserve cells (Liu et al. 2014; Stewart and Crook 2016; Moshi et al. 2020; Hopman et al. 2020). One of the first steps in the carcinogenic process in the uterine cervix is HPV infection of the basal cells of (metaplastic) squamous epithelium, reserve cells underlying the columnar epithelium in the transformation zone or cuboidal squamocolumnar junctional (SCJ) cells in the endocervix, after which SOX2-positive squamous CIN lesions arise. There is a consensus that SOX2 expression increases during the development of cervical cancer (Liu et al. 2014; Stewart and Crook 2016; Kim et al. 2015; Chang et al. 2015; Moshi et al. 2020), but in high-grade lesions SOX2 expression is not always found throughout the full thickness of the epithelium, while microinvasive cancer cells adjacent to CIN3 have been reported to be negative Stewart and Crook 2016. These authors suggest that progression of cervical squamous neoplasia may involve cyclical alterations in SOX2 activity. SOX2 biochemical analyses and tissue microarray studies are not always in line with the presented histological data, because the heterogeneity in SOX2 expression occurring throughout the tissue will not be detected by these techniques (Liu et al. 2014; Kim et al. 2015; Chang et al. 2015).

In this study, we focused on the histo-topographical distribution patterns of SOX2 immunostaining, in particular on its expression levels in the basal/parabasal, intermediate and superficial compartments of the CIN lesions. These SOX2 expression patterns will be correlated with the genetic make-up and viral load in the different epithelial compartments during subsequent stages of CIN.

## Materials and methods

### Tissue material

For this retrospective study of formalin-fixed and paraffin-embedded (FFPE) tissue, 57 patient samples were available; in 4 samples, no CIN lesion was detected, resulting in 53 cases that could be used in our study. Tissue samples, were collected between 2003 and 2009 and were selected from the archives of the Pathology Departments of the Maastricht University Medical Center Maastricht, The Netherlands (41 patients) and the Reinier de Graaf Hospital Delft, the Netherlands (16 patients).

Most samples were obtained by loop excision of the transformation zone (LETZ) to remove CIN. Prior to colposcopy, samples were obtained for cytological diagnosis (Maastricht series; 41 patients). Samples were analysed by multiplex ligation-dependent probe amplification (MLPA) for HPV type and viral load (Litjens et al. 2013). The samples for which no HPV data were available, the subtype type was determined on DNA isolated from the tissue section (Delft series). The final patient group included 10 cases of CIN1, 15 cases of CIN2, 28 cases of CIN3 as the final diagnosis and 4 cases showed no preneoplastic lesion. In total 153 areas were classified, including normal epithelium and coexisting CIN areas with a lower grade. The notations CIN1-2 and CIN2-3 refer to coexisting areas in which we could not unequivocally distinguish between CIN1 or CIN2 and CIN2 or CIN3, respectively.

Research on tissue and cell samples was performed in accordance with the Code for Proper Secondary Use of Human Tissue in the Netherlands (http://www.federa.org/) and was approved by the board of the Maastricht Pathology Tissue Collection at the Maastricht University Medical Centre (Registration Number MPTC 2011–05).

### Immunohistochemistry and fluorescence in situ hybridization

Immunohistochemical staining of 4 µm thick FFPE tissue sections was performed using primary antibodies against SOX2, p16 and Ki-67 (for detailed information on antibody characteristics,,detection methods and microscopy, see Supplemental Data, Table S1).

Immunohistochemical results were evaluated by means of bright field microscopy (153 areas in total) or fluorescence microscopy (5 normal areas and 5 areas within 5 samples for CIN1, 2 and 3 each). Brightfield microscopy of SOX2 expression was evaluated by visual inspection, taken into account staining intensity and distribution of the staining throughout the thickness of the epithelium. The Chi-square test was used as the statistical test because this test describes the statistical differences between categorical variables in expected and observed results (https://www.mathsisfun.com/data//chi-square-calculator.html).

Whole slide bright field TIF images were made using a Ventana iScan HT slide scanner (Roche, Ventana Medical Systems, Inc. Tucson, Arizona, USA). These scans were used as a roadmap to mark areas with different histological grade of CIN and to enable comparison of SOX2 expression patterns with fluorescence in situ hybridization (FISH) for HPV detection and analyses of the genetic make-up of different regions in the tissue.

To quantify SOX2 distribution throughout the epithelium, the fluorescent images for SOX2 expression were analysed by confocal laser scanning microscopy (Leica SPE confocal microscope; Amsterdam, the Netherlands) and ImageJ (Schindelin et al. 2012).

HPV subtypes were localized in the FFPE tissue sections using FISH or CISH with DNA probes for HPV 16, 18, 31 or 33 (PanPath, Budel, The Netherlands). For cytogenetic analysis, DNA probes were used for chromosomes 1, 3 and 7, and locus-specific probes for the genes *TERC, SOX2* and *SOX17* (for detailed information, see Supplemental Data and Table S2).

## Results

### SOX2 distribution patterns in normal squamous epithelia and CIN lesions

**Fig. 1:**
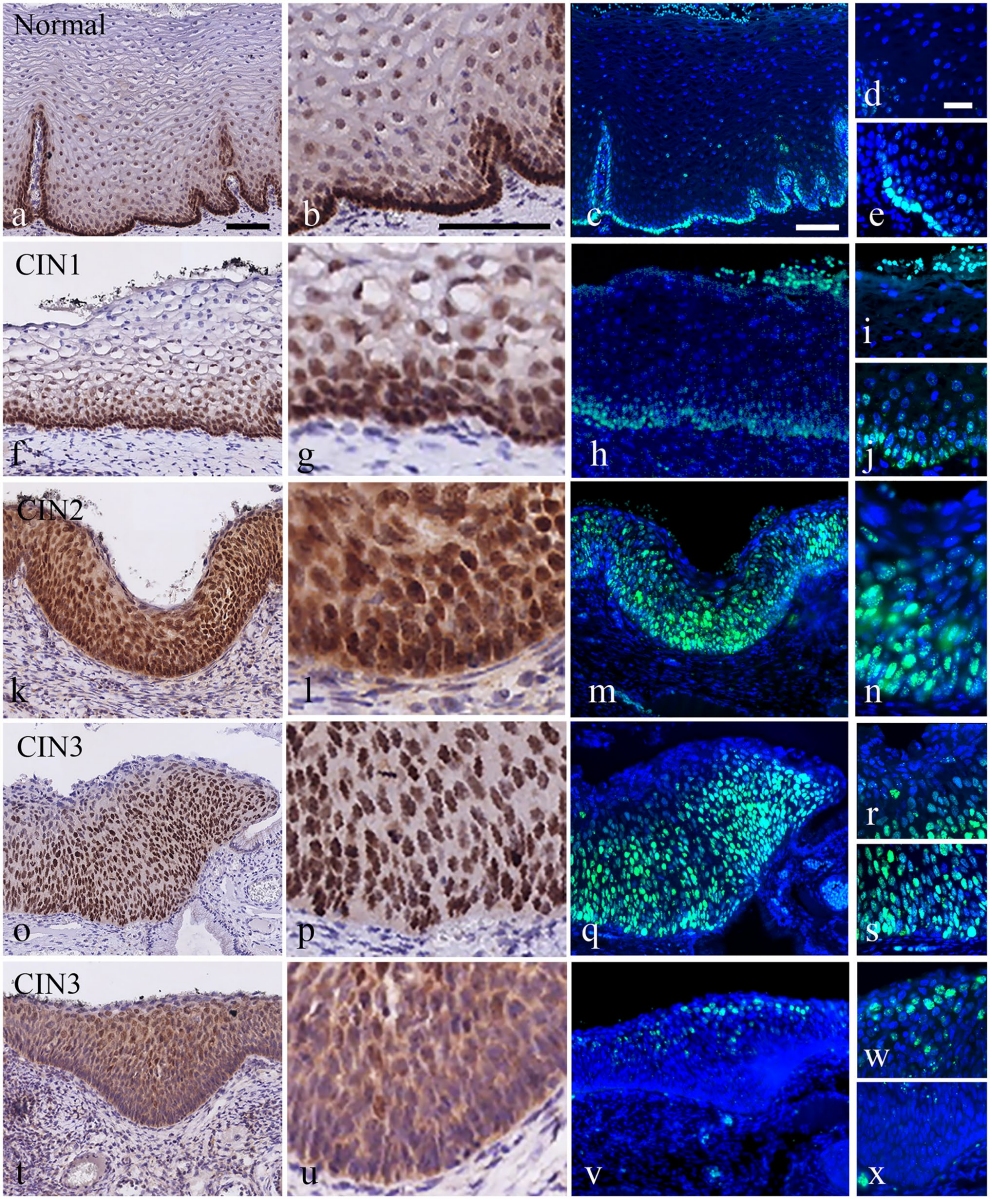
Comparison of SOX2 expression patterns in normal squamous epithelium and CIN1, CIN2 and CIN3 lesions as detected by bright field and fluorescence microscopy. **a**-**e** Expression of SOX2 in normal squamous epithelium in bright field microscopy **a**, **b**; higher magnification of a in **b** and fluorescence microscopy **c**-**e** with higher magnifications of superficial and basal layer showing no or very weak SOX2 expression in superficial cells **d** and strong expression in basal and parabasal cells **e**. **f**-**j** Expression of SOX2 in a CIN1 lesion with positive staining in basal and parabasal cells and moving gradually into the intermediate cell layer in bright field (**f**, **g**; higher magnification of **f** in **g**) and fluorescence microscopy (**h**-**j**). Higher magnifications clearly show the absence of SOX2 staining in the superficial area (**i**) and strong positivity in the basal and parabasal cell layers (**j**). The green fluorescence in **h** and **i** on top of superficial cells is caused by the high autofluorescence of erythrocytes in FFPE tissue. **k**-**n** Expression of SOX2 in a CIN2 lesion with strong positive staining throughout the squamous epithelium except for the superficial cells that were weakly positive in bright field (**k**, **l**; higher magnification of **k** in **l**) and fluorescence microscopy (**m**, **n**). The higher magnification in **n** clearly shows positive staining with a speckled pattern in the basal, parabasal and intermediate cell layers. **o**-**s** Expression of SOX2 in a CIN3 lesion in bright field microscopy (**o**, **p**; higher magnification of o in **p**) and fluorescence microscopy (**q**-**s**). Higher magnifications show a strong fluorescence in the basal/parabasal layer up to the intermediate layers (**r**) and a weaker/negative reaction in the more superficial cell layers (**s**). **t**-**x** Expression of SOX2 in a CIN3 lesion in bright field microscopy (**t**, **u**; higher magnification of **t** in **u**) and fluorescence microscopy (**v**-**x**), showing a negative reaction in the basal/parabasal and superficial cell layers, while showing SOX2 expression only in intermediate cell layers. Additionally, higher magnifications in w and x show that most epithelial cells are negative except for cells in the intermediate layers, which are positive with a speckled pattern. Scale bar = 100 μm in **a**, **b** and **c**. Magnifications in panels below a, b and c are similar. Scale bar = 20 μm in **d**; magnification in panels below **d** is similar

In the normal cervix, SOX2 is strongly expressed in the basal and parabasal layers of the squamous epithelium (Figs. 1a-e) and is absent in the endocervical columnar cells and in reserve cells (results not shown; see Moshi et al. 2020).

In the dysplastic epithelium, we distinguished 3 different SOX2 staining patterns (see also Table 1). In CIN1, the expression of SOX2 was found in the basal/parabasal cell layers and reached the intermediate cell layers (Figs. 1f-j). This pattern was classified as **Pattern 1** SOX2 staining. With progression of CIN the frequency of Pattern 1 decreased from 90% in CIN1 to 0% in CIN3 patients. In CIN2 and CIN3, SOX2 expression reached the superficial layers, and the intensity of expression increased compared to CIN1. This staining pattern was classified as **Pattern 2** and was seen in 44% of the CIN2 areas in CIN2 patients and 28% of the CIN3 areas in CIN3 patients (Figs. 1k-n and Figs. 1o-s, respectively). This staining pattern was associated with a strong immunostaining from the basal/parabasal compartment up to the superficial cell layers.

**Table 1:** SOX2 immunostaining and distribution patterns in normal cervical squamous epithelium and premalignant lesions

	Number of patients^a^	Histological classification of major and coexisting areas	Number of tissue areas^b^	Negative areas	SOX2 distribution patterns		
					Pattern 1	Pattern 2	Pattern 3
				*n* (%)	*n* (%)	*n* (%)	*n* (%)
		Normal^c^	53	3 (6)	50 (94)	0 (0)	0 (0)
LSIL	10	CIN1	10	1 (10)	9 (90)	0 (0)	0 (0)
HSIL	15	CIN2	18	1 (6)	6 (33)	8 (44)	3 (17)
		CIN1; CIN1-2	4	0 (0)	3 (75)	1 (25)	0 (0)
	28	CIN3	35	0 (0)	0 (0)	10 (28)	25 (72)
		CIN1; CIN1-2	12	1 (8)	10 (84)	1 (8)	0 (0)
		CIN2; CIN2-3	21	3 (14)	0 (0)	13 (62)	5 (24)
Total	*n* = 53		*n* = 153				

Different patterns of SOX2 staining were observed in normal and cervical interepithelial neoplasia (CIN1-CIN3). SOX2 staining patterns: Pattern 1) lower one-third: SOX2 was seen predominantly in the basal/parabasal layer and was weaker in the intermediate layer. Pattern 2) Lower two-thirds: SOX2 seen in the basal/parabasal and intermediate layers. Pattern 3) Upper one-third: SOX2 was seen only in the intermediate layer, with no or very low expression in the basal/parabasal layer

*LSIL* low-grade squamous intraepithelial lesion, *HSIL* high-grade squamous intraepithelial lesion

^a^Patients were classified on basis of the most severe histological area, coexisting areas with a lower grade in these patients are listed separately.

^b^All areas with the indicated histological classification were counted.

^c^Normal areas in patients classified as: no CIN lesion detected, CIN1, 2 or 3

Remarkably, in some CIN2 and most CIN3 patients, a SOX2 distribution pattern was recognized that deviated from that seen in Patterns 1 and 2, with a weak or no nuclear expression for SOX2 in the basal/parabasal compartment. The intermediate and superficial cell layers showed variable SOX2 expression in these cases (Figs. 1t-x). This pattern was classified as **Pattern 3**. In none of the low-grade CIN1 lesions this expression pattern was recognized, while the observed frequency of Pattern 3 increased from 17 to 72% in the areas CIN2 and CIN3, respectively. The frequency correlation of Patterns 2 and 3 with CIN2 and CIN3 demonstrated that Pattern 3 was strongly associated with CIN3 (*p* = 0.009; Chi-Square test).

**Fig. 2:**
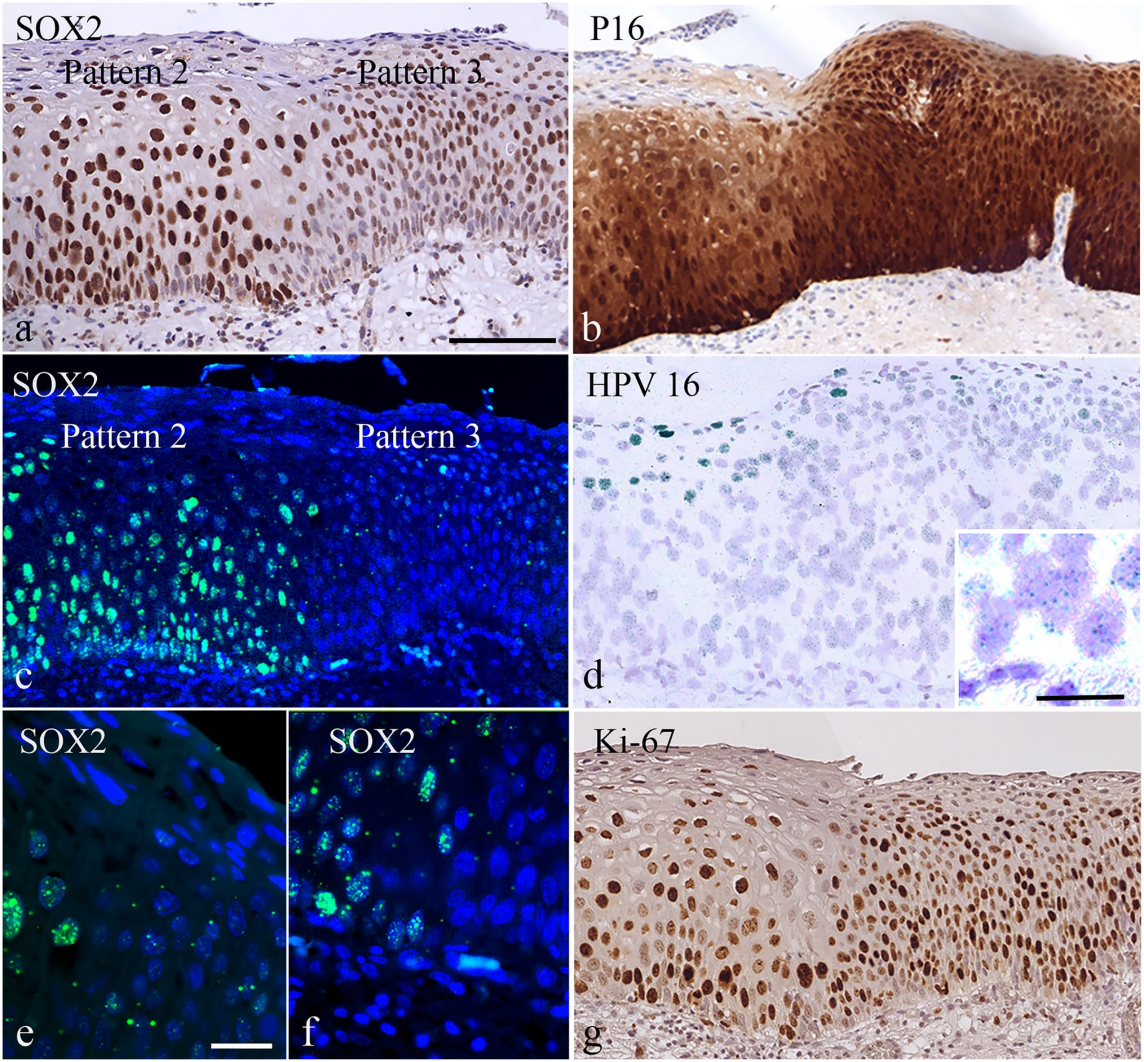
Transition area in a CIN3 lesion exhibiting adjoining Pattern 2 and Pattern 3 SOX2 distributions, with corresponding areas assessed for HPV and the proliferation marker Ki-67. (**a**, **c**, **e**, **f**) SOX2 expression level and distribution differences seen in Pattern 2 (left side) and Pattern 3 (right side) as visualized by bright field microscopy **a** and fluorescence microscopy **c**. Higher magnifications of the transition zone exhibit a speckled SOX2 pattern in individual cells (**e** and **f**). **b** P16 staining is strong in both Pattern 2 and Pattern 3, with no expression in the superficial layer in Pattern 2. **d** HPV load and physical status as detected by chromogenic in situ hybridization, showing viral replication in the superficial layer (productive lesion) and low viral load in the basal/parabasal nuclei in the Pattern 3 area (see insert). **g** Ki-67 expression in areas with SOX2 Pattern 2 and Pattern 3. Note the difference in nuclear size and the frequency of Ki-67-positive cells in the two different areas and that both areas harbor HPV. Scale bar = 100 μm in a. Magnification in panels **a**, **b**, **c**, **d** and **g** is similar. Scale bar = 20 μm in insert panel d and e. Magnification in **e** and **f** is similar

The distribution pattern of SOX2 staining throughout the dysplastic epithelium is sometimes complex, with a mixed or a discontinuous pattern. A typical example is depicted in Fig. 2, where CIN tissue exhibiting Pattern 2 adjoins tissue with Pattern 3 (see also Supplemental Figure S1).

Due to co-occurrence of areas with a different CIN grade within one and the same patient sample, also combinations of SOX2 expression patterns were observed in such tissue preparations. These coexisting lesions are included in Table 1. In 7 out of 28 CIN3 patients, Patterns 2 and 3 were coinciding simultaneously. In 12 areas with CIN1 or CIN1-2 and in 21 areas with CIN2 or CIN2-3, these lower grade areas the frequency distribution between Patterns 2 and 3 differed significantly (*p* = 0.002; Chi-Square test) from that seen in the CIN3 areas. Pattern 1 was detected in 10 out of the 12 coexisting CIN1 or CIN1-2 areas in these CIN3 patient samples. In summary, these data show that Pattern 1 is strongly associated with CIN1, Pattern 2 is predominantly detected in CIN2, while Pattern 3 is typical for CIN3.

### Quantification of SOX2 expression in the different staining patterns

**Fig. 3:**
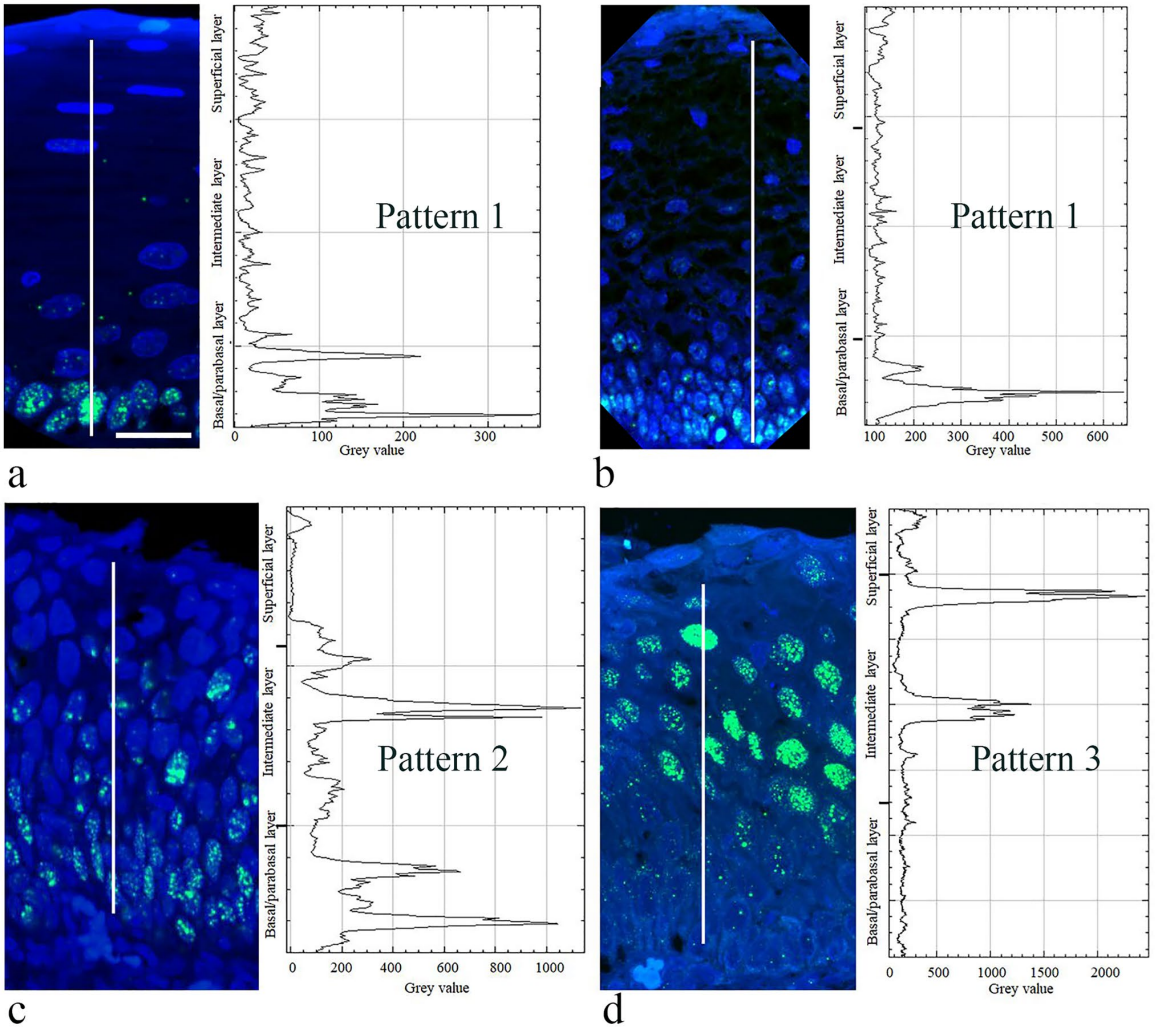
Quantitative analysis of SOX2 expression in areas with Patterns 1, 2 and 3. The expression levels of SOX2 were assessed by means of fluorescence intensity measurements in a confocal laser scanning microscope using a line scan (white line) in **a** normal squamous epithelium with SOX2 Pattern 1, **b** a CIN1 lesion with Pattern 1, **c** a CIN2 lesion with Pattern 2, and **d** a CIN3 lesion with Pattern 3. In Pattern 1, **a** high SOX2 fluorescence intensity is measured in the basal/parabasal cell layer only, while in Pattern 2, the fluorescence intensity moves gradually upwards to reach the intermediate cell layer. In Pattern 3, the basal/parabasal layer shows no SOX2 staining. Scale bar = 25 μm in **a**. Magnification in panels **a**, **b**, **c** and **d** is similar

The immunofluorescence intensity in the different patterns was quantified (Fig. 3 and Table 2) to obtain an objective interpretation of the SOX2 expression levels in CIN. Table 2 summarizes the distribution of the average SOX2 immunofluorescence intensities (and range) over the different cell layers quantified in the three different SOX2 distribution patterns. In Patterns 1 and 2, a high fluorescence intensity was measured in the basal/parabasal compartment, while in Pattern 3, the basal/parabasal compartment showed the lowest intensity. In Pattern 3, however, the nuclei in the intermediate layers showed the highest SOX2 expression levels, with a ten times higher fluorescence intensity compared to that of the basal/parabasal layers.

**Table 2:** Quantitative analysis of SOX2 expression levels in areas with Patterns 1, 2 or 3 as determined by means of fluorescence intensity measurements (Arbitrary Units)

		Basal/Parabasal cell layers	Intermediate cell layers	Superficial cell layers
Pattern 1	Average	341*	11	14
	Range	75–499	3–26	3–35
Pattern 2	Average	1767	264	27
	Range	547–3202	85–496	4–89
Pattern 3	Average	51	498	54
	Range	2–108	62–878	3–143

Fluorescence intensity was measured after an immunohistochemical staining procedure using FITC-tyramide as a peroxidase substrate. The SOX2 expression intensity was measured in 5 different sections. Each cell layer in a total of 50 nuclei was measured. Nuclei were not overlapping, nuclear truncation was minimal (large nuclear size) and on average 3.5 slices per nucleus (3–5 slices) were used for reconstruction. In total, 450 nuclei were reconstructed and measured (*50 nuclei per SOX2 pattern and per cell compartment). For quantification, the images are captured with the same fluorescence integration time during confocal microscopic imaging. The average fluorescence intensity and the range of fluorescence intensity are expressed in arbitrary units. ImageJ (NIH, Bethesda, Maryland, USA) was used for further image analysis, processing and merging/stitching of the fluorescent images for reconstruction of the sections

### Correlation of SOX2 distribution patterns with genetic aberrations in CIN lesions

**Fig. 4:**
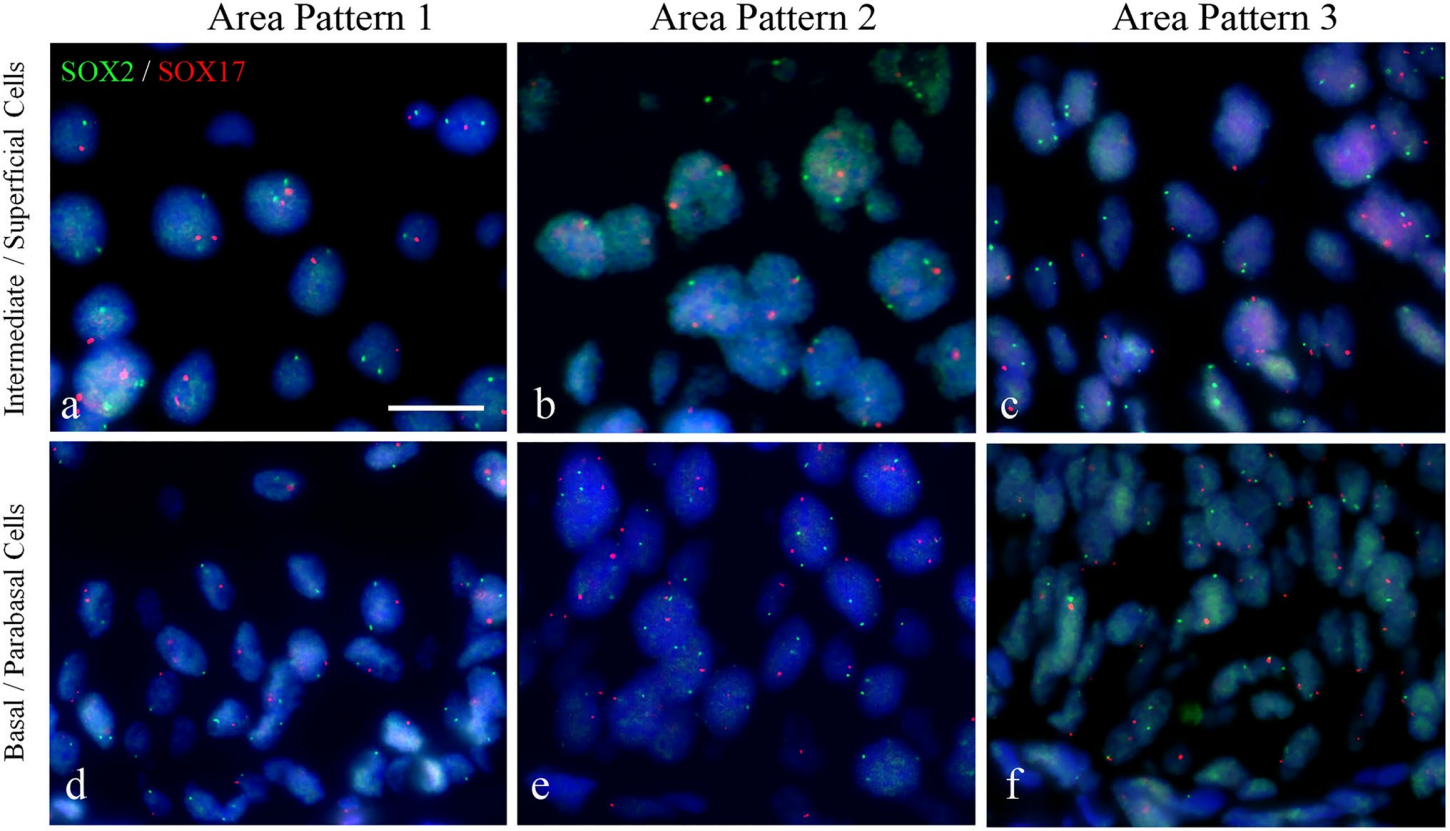
Fluorescence in situ hybridization showing copy number variations for the *SOX2* (visualized in green) and *SOX17* genes (visualized in red) in areas with SOX2 expression Patterns 1, 2 and 3. The intermediate and superficial cell layers are shown in **a**, **b** and **c**, while the basal/parabasal cell compartments are depicted in **d**, **e** and **f**. The Pattern 1 area was selected from a CIN1 lesion (**a**, **d**), while the Pattern 2 and Pattern 3 areas were from a CIN3 lesion (**b**, **c**, **e**, **f**). Genetically aberrant cells were mainly detected in areas with SOX2 Pattern 2, while in Patterns 1 and 3, the basal/parabasal cells were disomic. The intermediate layer in Pattern 3 showed genetically aberrant cells in most cases. Scale bar = 25 μm in **a**. Magnification in panels a-f is similar

*SOX2* (located at 3q26.32) copy number variations were analyzed in 95 tissue areas. No genetically aberrant cells were detected in the superficial layers of most CIN1 and CIN2 cases, (see Figs. 4a-c). Most CIN3 lesions, however, showed genetically aberrant cells in the intermediate/superficial compartment, with chromosomal copy numbers exceeding 2. Typical examples of FISH results in the basal/parabasal layer are shown in Figs. 4d-f, illustrating disomy for the *SOX2* gene in Pattern 1 (Fig. 4d), aberrant (aneusomic) cells in Pattern 2 (Fig. 4e) and disomy again in Pattern 3 (Fig. 4f). Table 3 summarizes the areas in which genetically aberrant cells were detected in the normal squamous epithelium and (pre)malignant lesions using FISH targeting the *SOX2* gene copy number. Several chromosomal probe sets were also tested and correlated with the different SOX2 patterns (see Supplemental Materials and Methods, Tables S2 and S3 and Figure S2).

**Table 3:** Presence of genetically aberrant (aneusomic) cells in the different cellular compartments of premalignant CIN lesions as detected by FISH targeting of *SOX2* copy numbers

Histological classification		SOX2 pattern	Number of areas^a^	Size of aberrant area	(Para)basal cell layers	Intermediate cell layers	Superficial cell layers
	Normal	1	29	Absent	N	N	N
LSIL	CIN1	1	16	Absent	N	N	N
		2	1	Small	A/N	A/N	N
HSIL	CIN1-2	1	2	Small	A/N	N	N
	CIN2	1	8	Medium	A	A/N	N
		2	5	Large	A	A	N
	CIN 2–3	2	4	Large	A	A	N
		3	4	Large	N	A/N	N
	CIN3	2	8	Large	A	A	N
		3	15	Large	N	A	A/N

*N* Normal (Disomy; copy number for *SOX2* = 2), *A*: Aneusomy (copy number for *SOX2* > 2), *A/N* Aneusomy/Normal (Aneusomic cells mixed with disomic cells), *LSIL* low-grade squamous intraepithelial lesion, *HSIL* high-grade squamous intraepithelial lesion. For a detailed description of the genetic classification, see the Supplemental Materials and Methods section. Note that Pattern 3 strongly deviated from Pattern 2. In all 19 cases, the SOX2-negative cells in the basal/parabasal cell layers were disomic for the *SOX2* gene, while the SOX2-positive intermediate cell layers showed aneuploidy for both targets in nearly all cases

^a^Not all cases mentioned in Table 2 were available for FISH analysis. Only the number of analyzed areas is indicated.

In summary, disomic cells were detected in all CIN1 lesions (Pattern 1), while genetically aberrant cells were detected in the (para)basal and intermediate compartments of the CIN2 and CIN3 lesions exhibiting Pattern 2. In Pattern 3, an unexpected distribution pattern of genetically aberrant nuclei was observed, with disomic nuclei in the basal/parabasal compartment (for the mentioned chromosomal probe sets). In contrast, in the majority of these cases, the intermediate compartment contained genetically aberrant nuclei.

### Correlation of SOX2 patterns with the presence of HPV

**Fig. 5:**
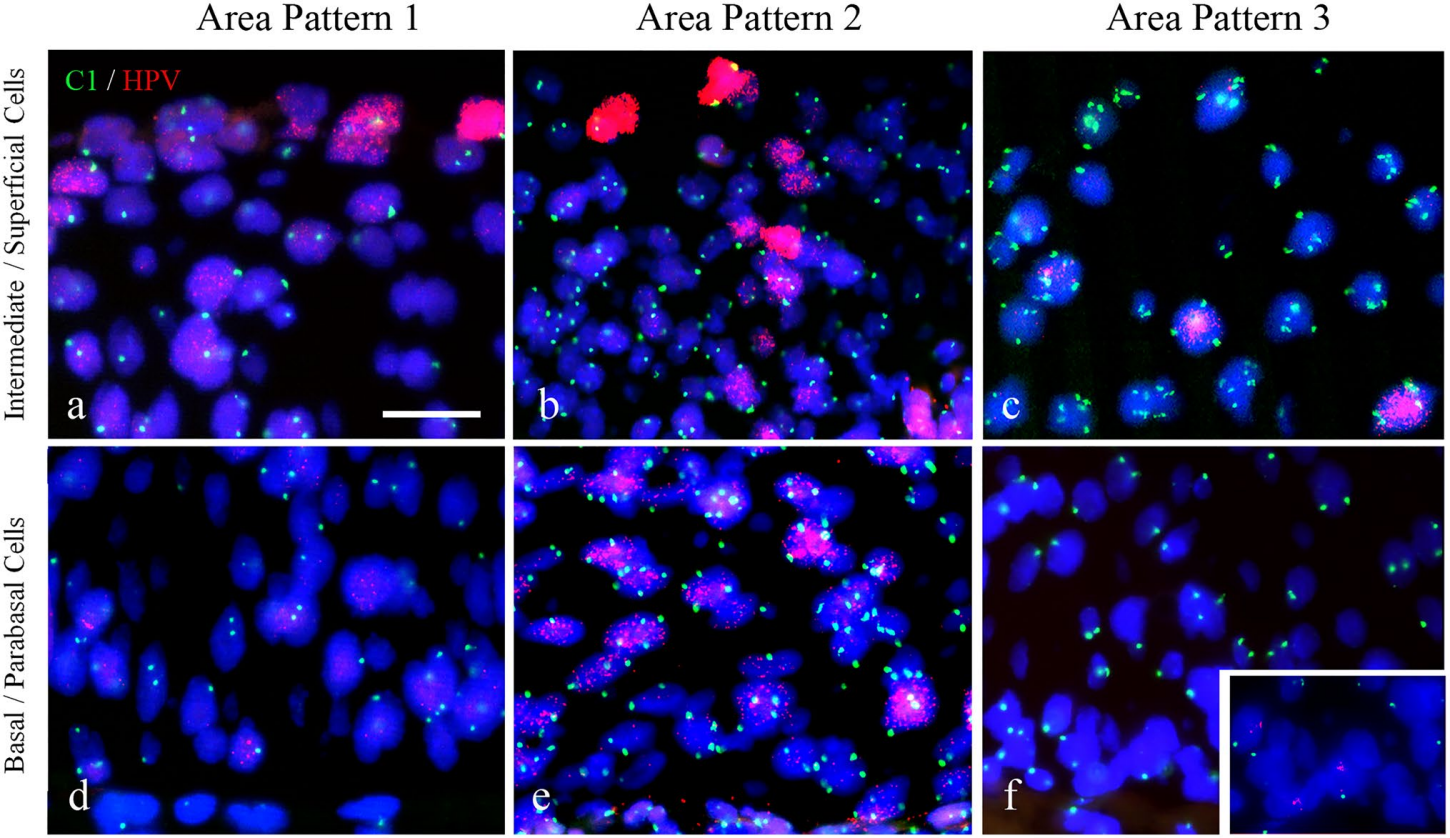
Fluorescence in situ hybridization targeting copy number variations for the centromere region of chromosome 1 (C1; visualized in green) and load for HPV 16 (visualized in red). The intermediate and superficial cell layers are shown in **a**, **b** and **c**, while the basal/parabasal cell compartments are depicted in **d**, **e** and **f**. The Pattern 1 area was selected from a CIN1 lesion (**a**, **d**), while the Pattern 2 and Pattern 3 areas were from a CIN3 lesion (**b**, **c**, **e**, **f**). Genetically aberrant cells were seen in areas with SOX2 Pattern 2, while in Patterns 1 and 3, the basal/parabasal cells were disomic. The intermediate layer in Pattern 3 showed genetically aberrant cells in most cases, with the highest viral load also seen in the intermediate/superficial cells showing replication of the viral sequences. In Pattern 2, a relatively high copy number for the virus is also seen in the basal/parabasal cells, while in Patterns 1 and 3, a low HPV copy number is seen in these basal/parabasal layers (see insert in **f**). Scale bar = 25 μm in **a**, Magnification in panels **a**-**f** is similar

The physical presence of HPV in the different cellular compartments could be correlated with the different SOX2 patterns. A typical example with a sharp delineation between SOX2 patterns is shown in Fig. 2. The panel illustrates the positive p16 staining and high proliferative activity in the basal/parabasal and intermediate cell layers on both sides of the delineation. In the low-grade lesions, HPV ISH revealed extensive viral replication (a productive viral infection) in the superficial layer (Fig. 2d and Figs. 5a, b) with a low viral load in the basal/parabasal compartment. In contrast, areas with SOX2 Pattern 3 showed p16 staining in all cell compartments of the lesion, with no clear HPV replication pattern in the superficial layers (Fig. 2d and Fig. 5c). The genetically normal basal/parabasal cell layers most strikingly contained a low HPV viral load (Table 4).

**Table 4:** Presence of genetically aberrant (aneusomic) cells in the different cellular compartments of (pre)malignant CIN lesions as detected by FISH targeting of chromosome 1 centromere (1C) copy numbers

Histological classification		SOX2 pattern	Number of areas^a^	Size of aberrant area	(Para)basal cell layers	Intermediate cell layers	Superficial cell layers
LSIL	CIN1	1	10	Small	N	N	N
		2	1	Small	A/ N	A/ N	N
HSIL	CIN1-2	1	3	Small	A/ N	N	N
	CIN2	1	14	Small	A	A/ N	A/ N
		2	3	Medium	A	A	A/ N
	CIN2-3	2	4	Large	A	A	A/ N
		3	4	Large	N	A	A/ N
	CIN3	2	9	Large	A	A	A/ N
		3	12	Large	N	A	A/ N

*N* Normal (Disomy; copy number for centromere of chromosome 1 (1C) = 2), *A* Aneusomy (copy number for centromere of chromosome 1 (1C) > 2), *A/N* Aneusomy/Normal (Aneusomic cells mixed with disomic cells). For a detailed description of the genetic classification, see the Supplemental Materials and Methods section. All histologically normal areas were genetically normal (disomic) and p16 negative. *LSIL* low-grade squamous intraepithelial lesion, *HSIL* high-grade squamous intraepithelial lesion

^a^Not all cases mentioned in Table 2 were available for FISH analysis. Only the number of analyzed areas is indicated.

#### Discussion

**Fig. 6:**
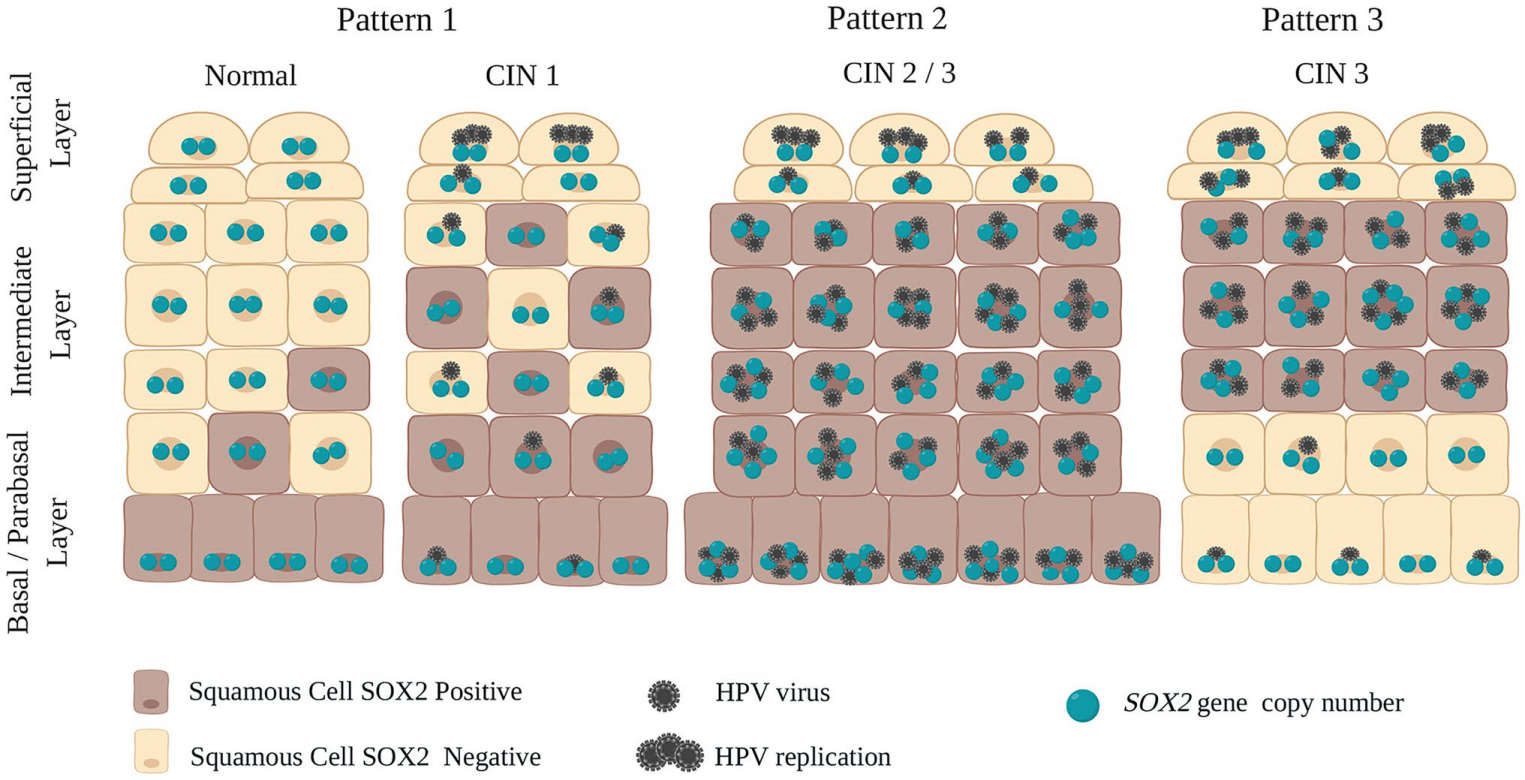
Schematic representation of the different SOX2 distribution patterns, with concomitant molecular characteristics, for the normal squamous epithelium and the different stages of cervical premalignant lesions

Cervical intraepithelial neoplastic (CIN) lesions are SOX2 positive, with increased expression of this transcription factor upon progression from normal/CIN1 to CIN3. Three SOX2 distribution patterns can be recognized that type the different stages of CIN. Pattern 1 is strongly associated with CIN1, while Pattern 2 is detected in most CIN2 and some CIN3. These data for high-grade squamous intraepithelial lesions (HSIL) support previous studies that reported SOX2 staining throughout the full thickness of the epithelium (Kim et al. 2015; Chang et al. 2015; Stewart and Crook 2016). In CIN3, however, we observed a strongly deviating pattern (Pattern 3) characterized by the absence or low expression of SOX2 in the basal/parabasal compartment and variable levels in the intermediate and superficial compartments (for a schematic representation, see Fig. 6). SOX2 negative (areas in) high-grade squamous intraepithelial lesions (HSIL) have been reported before (Stewart and Crook 2016), while also downregulation of SOX2 was seen during the initial stages of the invasive process (Stewart and Crook 2016, 2018).

The absence or low SOX2 expression in Pattern 3 prompted us to study the molecular characteristics of the different epithelial layers in these high-grade premalignant lesions more carefully. The genetic make-up of CIN has been studied in detail during progression of these premalignant lesions, and it was shown that genetic instability increased during progression of CIN (Heselmeyer et al. 1996; Rao et al. 2004; Hopman et al. 2004, 2006; Foster et al. 2009; Koeneman et al. 2019).

There is no clear-cut correlation between the gene dose for SOX2 and its level of expression. On the one hand, one would expect such a correlation to exist in the “Pattern 3 lesions” when correlating the *SOX2* gene copy numbers to SOX2 expression levels, i.e. > 2 copies in SOX2 positive intermediate layers and 2 copies in the SOX2 negative (para)basal layers. However, SOX2 expression is also found in (para)basal cell layers of normal squamous epithelium and CIN1 with only 2 copies of the *SOX2* gene. Most likely, the expression of SOX2 in these cell compartments is correlated to the stemness of the normal basal cell compartment, but also to enhanced tumorigenicity in the CIN3 lesion (Liu et al. 2014), and as shown before for other (pre)malignancies (for a summary see Mamun et al. 2020). SOX2 expression gain may also result via the observed 3q26 chromosomal amplification. Since 3q26 copy number gains are a frequently occurring mutation in cervical carcinomas, it might be involved in the (over)expression of SOX2 and ultimately lead to the establishment of a stem-like, tumor-initiating cell phenotype.

On the basis of the finding that the basal/ parabasal compartment in a majority of CIN3 lesions shows a normal genetic make-up and the observation of direct morphological transitions between (atypical) immature metaplasia and CIN3 with concomitant molecular switches, we propose three different models for the route of HPV infection and the origin and progression of CIN lesions.

**Fig. 7:**
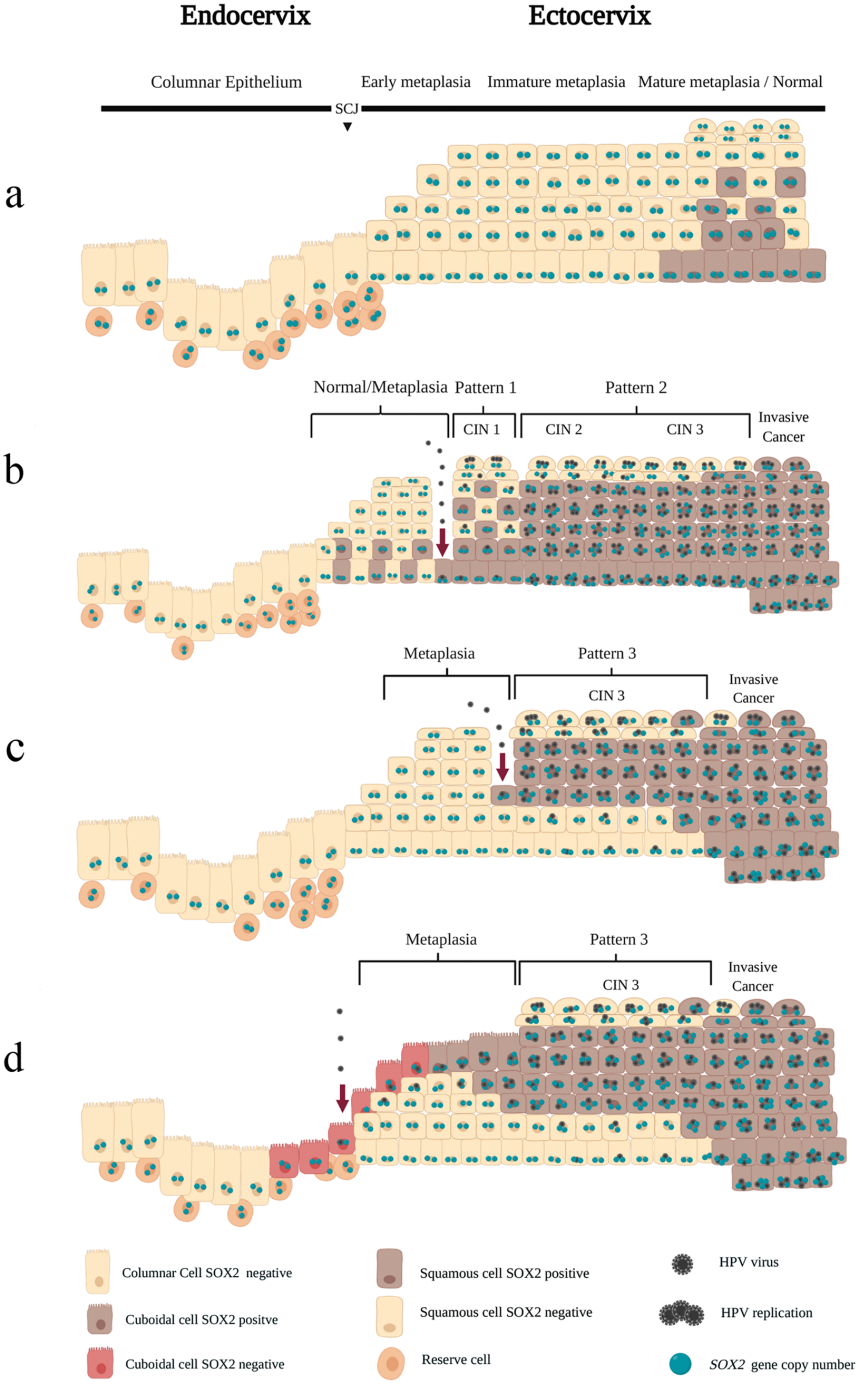
Schematic representation of the different models for the origin of preneoplastic CIN lesions after HPV infection (indicated by the arrow) based on the different SOX2 expression patterns and genetic characteristics. **a** Origin of metaplastic epithelial changes and increasing SOX2 expression with a higher degree of maturation. **b** HPV infection of the basal cell layer(s) in the normal or metaplastic epithelium of the transformation zone results in CIN1, which progresses to invasive squamous cell carcinoma via CIN2 and CIN3. **c** HPV infection of the intermediate cell layers in the normal or metaplastic epithelium of the transformation zone resulting directly in CIN3, subsequently progressing to invasive squamous cell carcinoma. **d** HPV infection of the columnar cell layer overgrowing the normal or metaplastic epithelium of the transformation zone (as suggested by Herfs et al. 2012, 2013) results directly in CIN3, subsequently progressing to invasive squamous cell carcinoma

In the following paragraphs, we will discuss different progression models for cervical carcinogenesis (Figs. 7a-d) that can explain the molecular make-up of CIN3, taking into account the literature on the progression of CIN lesions (Woodman et al. 2007; Chen et al. 2010; Herfs et al. 2012, 2013; Bosch et al. 2013; Litjens et al. 2014; Doorbar et al. 2015; Mirkovic et al. 2015; Mills et al. 2017; Moshi et al. 2020; Hopman et al. 2020).

### Model 1: HPV infection of the (para)basal cell layers in normal squamous epithelium or mature metaplasia results in CIN1, which progresses to CIN2 and CIN3 (Figs. 7a, b).

The conventional model for the carcinogenic process in the uterine cervix, as proposed by Woodman et al. 2007 and Bosch et al. 2013, they suggest that HPV infection of the basal cells of the squamous epithelium or in mature squamous metaplasia initiates a cascade of events resulting in CIN1 (see also Herfs et al. 2012), which in a minor fraction of patients progresses to invasive squamous cell carcinoma (SCC) via CIN2 and CIN3 (Litjens et al. 2014; Mills et al. 2017).

Most CIN1 lesions have been described to originate from infected (para)basal cells in the ectocervix (Mirkovic et al. 2015). Our observation that the SOX2-positive basal cell compartments of normal and mature metaplastic epithelium (Fig. 7a) show a SOX2 expression pattern similar to that of CIN1 (Pattern 1), with a subsequent increase in SOX2-positive cell layers with increasing severity of the lesion (Fig. 7b), indeed supports the suggestion that such a sequence of events can take place during progression of cervical preneoplasia. The observation that some of the CIN3 lesions show a SOX2 expression pattern similar to that of most CIN2 lesions suggests that at least this part of the high-grade lesions develops through progression from CIN2.

### Model 2: HPV infection of the intermediate cell layers in immature metaplasia results in CIN3 (Fig. 7c)

A significant fraction (72% of CIN3 areas) of the CIN 3 patients showed the typical SOX2 Pattern 3, together with Pattern 2, which is typical for CIN2. Pattern 3 is characterized by low or no expression of SOX2 in the (para)basal cell compartments and more extensive staining in the intermediate cell layers upwards. The fact that these (para)basal cell layers are SOX2 negative or weakly positive indicates that they may be part of immature metaplasia (Moshi et al. 2020). In the underlying study, the basal compartments in a high frequency of CIN3 lesions were shown to exhibit a normal genetic make-up as defined by ploidy for chromosomes 1 and 3 and copy numbers for the SOX2 and SOX17 genes. This suggests that an HPV infection could have occurred in the intermediate cell layers in the normal or metaplastic epithelium in the transformation zone, resulting directly in CIN3, omitting the involvement of lower grade lesions as precursors.

This model is supported by literature reports indicating that CIN3 lesions are rarely preceded by a CIN1 lesion (Chen et al. 2010; Herfs et al. 2012; Mirkovic et al. 2015; Mills et al. 2017). The majority of metachronous CIN1 and CIN3 lesions, for example, caused by different HPV genotypes, indicating that a progressive biologic continuum from CIN1 via CIN3, leading finally to cervical cancer, may be unlikely in at least some cases (Litjens et al. 2014). In addition, the SOX2 immunostaining results for Pattern 3 suggest a relatively rapid development of a high-grade lesion without a well-defined low-grade state. Indeed, Woodman et al. 2007 showed that detecting high-grade CIN was maximal 6 to 12 months after the first detection of HPV16.

### Model 3: HPV infection of the cuboidal squamocolumnar junction cells results in CIN3 (Fig. 7d)

HPV infection of a discrete population of cuboidal squamocolumnar junctional (SCJ) cells with a unique morphology and gene-expression profile has been suggested by Herfs et al. 2013 and Mirkovic et al. 2015 to result in a premalignant squamous cervical lesion. The biomarker expression profile typical for these cells was also detected in a high percentage of high-grade CIN lesions.

Additionally, this model, suggesting a downward rather than an upward evolution from progenitor cells to the premalignancy, could explain the observation by Chen et al. 2010; Litjens et al. 2014 and Mills et al. 2017 that CIN3 can originate without previous precursor lesions. HPV infection of the squamocolumnar junction (SCJ) cells was suggested to result in a trans-differentiation process with an outgrowth of subjacent squamous cells (so-called top-down differentiation), often leading to high-grade lesions (Herfs et al. 2013). In the model proposed in Fig. 7d, HPV-infected SCJ cells overgrow the normal squamous epithelium and initiate a downward proliferation and differentiation to SOX2-negative (immature) squamous metaplasia, which further develops into CIN3, proliferating in an upward direction. This model explains not only the normal genetic make-up of the (para)basal cells but also the aneuploidy and viral load detected in the intermediate cell layers.

### Is the absence of SOX2 expression and a normal genetic make-up in the (para)basal cell layers an indication for regression of the CIN3 lesion?

As described by Doorbar et al. 2015 lesion regression, when it does occur after HPV infection, is not associated with significant apoptosis or cell death. Animal model studies have shown that lesions are cleared by the replacement of actively infected cells with apparently normal basal cells that continue to divide These histologically normal cells can still contain viral genomes but without evident viral gene expression. The characteristics of the (para)basal cells in Pattern 3 answer to a certain extent to these properties in that they are p16 positive, proliferating and show a low HPV load but exhibit an apparently normal genomic composition.

If Pattern 3 is specific for a regressive process, it could be expected that this SOX2 distribution pattern should be detected in the majority of CIN1 lesions. However, the cells targeted by HPV in these low-grade lesions are the basal cells shown to be SOX2 positive, while in the formation of higher-grade lesions, HPV infection occurs in SOX2-negative immature metaplasia.

We conclude that our data shed new light on the biological characteristics and dynamics in the development of premalignant cervical lesions, as well as on the initial viral infection of the target cells for HPV in the area of the squamocolumnar junction. SOX2 staining patterns may become a useful marker to distinguish CIN3 from low-grade CIN, particularly in diagnostically challenging cases.

## Supplementary Information

Below is the link to the electronic supplementary material.

Supplementary file1 (PDF 133 kb)

Supplementary file2 (TIF 7512 kb)

Supplementary file3 (TIF 7285 kb)

## Data Availability

The study includes original data, A.H.N. Hopman confirms that he had full access to all the data in the study and takes responsibility for the integrity of the data and the accuracy of the data analysis.
