# Supplementary material for: SOX2 expression in the pathogenesis of premalignant lesions of the uterine cervix: its histo-topographical distribution distinguishes between low- and high-grade CIN
Source: Histochem Cell Biol. 2022 Aug 9;158(6):545–59. doi: 10.1007/s00418-022-02145-6 (PMC9726813; doi:10.1007/s00418-022-02145-6)
Supplement: Supplementary file 1 — Supplementary file1 (PDF 133 kb) [file 418_2022_2145_MOESM1_ESM.pdf]

Supplemental data

Supplemental Materials and Methods

SOX2 expression in the pathogenesis of premalignant lesions of the uterine cervix: its histo-topographical distribution distinguishes between low and high grade CIN.

Jobran M. Moshi[1, 2], Monique Ummelen[1], Jos L.V. Broers[1], Frank Smedts[3], Koen K. Van de Vijver[4,5], Jack P.M. Cleutjens[4], Rogier J.N.T.M. Litjens[6], Frans C.S. Ramaekers[1], Anton H. N. Hopman[1, *]

[1]Department of Molecular Cell Biology, GROW-School for Oncology and Reproduction, Maastricht University Medical Center, Maastricht, the Netherlands.
[2]Department of Medical Laboratory Technology, Faculty of Applied Medical Sciences, Jazan University, Jazan, Kingdom of Saudi Arabia.
[3]Department of Pathology, Cork University Hospital, Cork, Ireland.
[4]Department of Pathology, CARIM-Cardiovascular Research Institute Maastricht, Maastricht University Medical Center, Maastricht, the Netherlands.
[5] Department of Pathology, Ghent University Hospital, Cancer Research Institute Ghent (CRIG), Ghent, Belgium.
[6]Department of Gynecology, Laurentius Hospital Roermond, The Netherlands.

*Corresponding author:
Anton Hopman, Ph.D.
Department of Molecular Cell Biology,
Maastricht University Medical Center,
P.O. Box 616, 6200 MD Maastricht, the Netherlands.
Mobile: +31 646139090.
E-mail: hopman@maastrichtuniversity.nl

Immunohistochemistry

After standard deparaffinization in xylene and blocking of endogenous peroxidase activity by 0.3% H_2O_2 in methanol, the 4 µm thick FFPE tissue sections were boiled in 10 mM Tris-EDTA buffer pH 9.0 for 20 min in a microwave oven for antigen retrieval and incubated at room temperature to cool down. For bright field detection as well as fluorescent detection the same primary and secondary antibodies and enhancement kits were used as listed in Supplemental Table S1.

For bright field microscopy the antigens were detected with the primary antibodies, followed by secondary antibodies and enhancement procedures, stained with diaminobenzidine.HCl (DAB) and finally counterstained with hematoxylin for bright field microscopical examination. It is important to note that dilution of the primary SOX2 antibody, in combination with a sensitive secondary antibody, was optimized to obtain a positive nuclear staining throughout the epithelium. This could result in some cytoplasmic background staining in the endocervical epithelium.

For the fluorescent detection of SOX2 the catalyzed reporter deposition (CARD) reaction was performed as described earlier using FITC-tyramide as a substrate (AlexaFluor488 tyramide reagents, Invitrogen, Fisher Scientific, Merelbeke, Belgium). The tyramide reaction at a concentration of 10 ug/ml was performed in PBS containing 0.1 M imidazole, pH 7.6, and 0.001% H_2O_2, for 15 minutes at room temperature. The slides were washed with MilliQ water, dehydrated in an ascending ethanol series and mounted in Vectashield (Vector Laboratories), containing 4',6-diamidino-2-phenylindole (DAPI; Sigma; 0.5 µg/ml) for nuclear DNA staining.

Imaging of bright field and fluorescent immunostaining

The bright field sections were scanned with a Roche Ventana iScan HT slide scanner (Roche, Ventana Medical Systems, Inc. Tucson, Arizona, USA) and semi-quantitatively scored for expression of SOX2 and p16. Images were viewed and selected using Image Viewer Software (Roche, Ventana MS).

The fluorescently labelled sections were imaged with a laser scanning confocal microscope (Leica SPE confocal) using LAS AF software (Leica Application Suite Advanced Fluorescence, version 2.7.3.9723). Imaging was done in acquisition mode xyz, with an ACS APO 63.0x1.30 oil immersion lens, a gain of 800-1000V, offset between -0.5 and 0%, format of 1024x1024 pixels, speed of 400 Hz and frame average of 3. For excitation, a laser wavelength of 405 nm (approx. 10% power) was used (pre-defined LUTs, GLOW O&U).

FITC emission was recorded at 488nm (detection bandwidth 5nm) in acquisition mode xyz. Images were imported in Image J, NIH, Bethesda, Maryland, USA) and areas of interest were selected. The scans from the confocal microscope were combined (summed) and the channels were split in red, green, and blue. A fluorescent line scan measurement was performed to obtain a profile plot across the epithelial layers of interest (basal to superficial layers). The intensity curve was used to set the background correction. The background was subtracted from the total fluorescence intensity measured. For the total fluorescence intensity per nucleus, the blue DNA staining (DAPI image) was used to set the contour of the measurement. For each SOX2 pattern 5 different patients were analyzed, and per area 30 nuclei (10 in the basal/parabasal layers, 10 in the intermediate layers and 10 in the superficial layers) were measured.

In Situ Hybridization

Fluorescence (FISH) or chromogenic in situ hybridization (CISH) for HPV detection:

The probes for HPV16, HPV18, HPV31 and HPV33 (PanPath, Uden, The Netherlands) were labelled with biotin by standard nick translation and used at a concentration of 1 ng/µl in a solution containing 50% formamide, 2×SSC (saline-sodium citrate buffer; 0.15 M sodium chloride and 15 mM trisodium citrate pH 7.0), 10% dextran sulphate (Sigma, Steinheim, Germany; MW>100,000) and 50 times excess of carrier DNA (salmon sperm DNA; Sigma, Steinheim, Germany). The HPV probes were applied under a coverslip, denatured for 10 min at 80°C and hybridized overnight at 37°C. After the post-hybridization washings for FISH the hybridized HPV-biotin probes were detected with a triple layer detection method, consisting of subsequently FITC or TRITC-conjugated avidin (1:500 dilution; Vector Laboratories, CA, USA), biotinylated goat anti-avidin (1:100 dilution; Vector Laboratories) and finally FITC or TRITC-conjugated avidin, each for 15 min at RT. All incubations were performed in 4x SSC containing 5% non-fat dry milk. The slides were washed in 4x SSC containing 0.05% Tween-20, dehydrated in an ascending ethanol series and mounted in Vectashield (Vector Laboratories), containing 4',6-diamidino-2-phenylindole (DAPI; Sigma; 0.5 µg/ml).

For CISH, the hybridized HPV probe was detected in a triple layer detection method with subsequently peroxidase-conjugated avidin (1:100 dilution; Vector Laboratories, CA, USA), biotinylated goat anti-avidin (1:100 dilution: Vector Laboratories) and finally peroxidase-conjugated avidin. The enzymatic reaction was performed using Vina Green according to the instructions of the supplier (Vina Green Chromogen Kit, BIOCARE Medical, CA, USA).

Finally, the slides were washed in milliQ, counterstained with hematoxylin, immediately dehydrated and coverslipped using Entellan new (Merck, Darmstadt, Germany).

Fluorescence in situ hybridization (FISH) to detect chromosomal DNA targets:

The following chromosomal probes were used: the chromosome 1 centromere (1C) probe 1q12, the chromosome 3 centromere (3C) probe pα3.5, the chromosome 7 centromere (7C) probe p7t1, the *SOX2* probes *3q26*.3 (RP11-252018 and RP11-9205), the *TERC* probes *3q26*.1–2 (RP11-1141L22 and RP11-990E14) and the *SOX17* probes *8q12* (RP11-30G11 and RP11-235L19). As indicated in Supplemental Table S2, these probes were labelled with either biotin, digoxigenin or Atto425 in a nick translation labelling procedure (Jena Bioscience GmbH, Jena, Germany).

The probes were hybridized at a concentration of 2 ng/µl (1C), 3 ng/µl (3C), 2 ng/µl (7C), 5 ng/µl (*TERC*), 5 ng/µl (*SOX2*) and 5 ng/µl (*SOX17*). The probes were incubated together with 10 times excess COT1 DNA (Thermo Fisher Scientific, Waltham, Massachusetts, United States), and 75 times excess of carrier DNA (salmon sperm DNA; Thermo Fisher Scientific) in a mixture of 50% formamide (Sigma, Steinheim, Germany), 2x SSC (Sigma) and 10% dextran sulphate Mw>100.000 (Sigma)

The biotin labelled probes were detected in green with a triple layer detection method using FITC conjugated avidin as described above for the HPV probes. The digoxigenin labelled probes were detected in red with a mouse monoclonal anti-digoxigenin antibody (MaDig, 1:100 dilution, Sigma, St Louis MO, USA), followed by rabbit anti-mouse-TRITC (RaM-TRITC, 1:100 dilution, Dako/Agilent, Glostrup, Denmark) and swine anti-rabbit-TRITC (SwaR-TRITC. 1:100 dilution, Dako). For Atto425 labelled probes (blue detection) no further immunologic detection is needed and the probe could be co-hybridized with biotin and digoxigenin labelled probes. For the bi-color detection with biotin and digoxigenin labelled probes the triple layer detection methods were combined. For the triple-color detection method the Atto425 probe is added to the hybridization mix. For an overview of the combinations of DNA probes used for FISH detection of copy number variations see Supplemental Table S3.

Imaging of FISH and CISH signals

CISH images were recorded with the Nikon Eclipse E800 (mounted with a color camera) and Nikon ACT-1 software. FISH images were recorded with the Metasystems Image Pro System

(black and white CCD camera; Sandhausen, Germany) mounted on top of a Leica DM-RE fluorescence microscope, equipped with TRITC, FITC and DAPI single band pass filters for single color analysis using an APO 63.0x1.30 oil immersion lens. Images were obtained using an automatic integration time, allowing semiquantitative evaluation, and using the full dynamic range of the camera without signal intensity saturation To visualize the low copy numbers of HPV signals a long integration time for fluorescence in situ capturing was needed. Frequently imaging was complicated by the autofluorescence of FFPE tissue section.

Supplemental Table S1:
Antibody characteristics and optimized immunohistochemical detection methods.

Antigen	Primary Antibody	Dilution	Secondary antibody	Enhancement/Detection
SOX2	mAb Rabbit IgG<br>clone EPR3131<br>Ab92494<br>Abcam,<br>Cambridge, UK	1:100 in<br>PBST/45<br>min, 37^{0}C<br>5% NGS	Biotinylated Goat anti Rabbit IgG, BA-1000, Vector Laboratories, Burlingham, CA, USA<br>1:200 in PBST/5% NGS<br>30 min, 37^{0}C	ABC, Vectastain Elite ABC Kit,<br>Vector Laboratories, Burlingham, CA, USA, 30 min RT; DAB reaction or CARD reaction using FITC-tyramide, Fisher Scientific, Merelbeke, Belgium.
p16	mAb Mouse IgG2a<br>Clone E6H4<br>CINtec, MTM Laboratories AG, Heidelberg, Germany	1:50 in<br>PBST/1%<br>BSA,<br>1 hr RT	Biotinylated Horse anti Mouse IgG, BA-2001, Vector Laboratories, 1:200 in PBST/1% BSA; 30 min RT	ABC<br>30 min RT,<br>DAB reaction
Ki-67	mAb mouse IgG1<br>Clone MIB-1<br>M7240<br>Dako/Agilent, Glostrup, Denmark	1:25 in<br>PBST/1%<br>45 min,<br>37^{0}C<br>BSA	Biotinylated Horse anti Mouse IgG, BA-2001<br>Vector Laboratories,<br>1:200 in PBST/1% BSA<br>30 min, 37^{0}C	ABC<br>30 min RT,<br>DAB reaction

Abbreviations: mAb, monoclonal antibody; HRP, horseradish peroxidase; RT, room temperature; PBST, phosphate buffered saline + 0.1% Tween-20 (Janssen Chimica, Beerse, Belgium); BSA, bovine serum albumin; DAB, diaminobenzidine; ABC, Avidin-biotin complex with biotinylated HRP; NGS, normal goat serum.

Supplemental Table S2:

Summary of labelled HPV and chromosomal DNA probes used for direct and indirect fluorescent detection using in situ hybridization (FISH).

Probe name	Label	Fluorescent detection
HPV (16, 18, 31 or 33)	Digoxigenin	Red: MaDig, RaM-TRITC, SwAR-TRITC
1C Chromosome 1 centromere: 1q12	Atto425	Blue: No immunological detection needed
1C Chromosome 1 centromere: 1q12	Biotin	Green: Av-FITC, Bio-GaA, Av-FITC
3C Chromosome 3 centromere: pα3.5	Atto425	Blue: No immunological detection needed
3C Chromosome 3 centromere: pα3.5	Digoxigenin	Red: MaDig, RaM-TRITC, SwAR-TRITC
7C Chromosome 7 centromere: p7t1	Digoxigenin	Red: MaDig, RaM-TRITC, SwAR-TRITC
TERC Telomerase RNA component: *3q26*.1–2 (RP11-1141L22 and RP11-990E14)	Biotin	Green: Av-FITC, Bio-GaA, Av-FITC
SOX2 3q26.3 (RP11-252018 and RP11-9205)	Biotin	Green: Av-FITC, Bio-GaA, Av-FITC
SOX17 8q12 (RP11-30G11 and RP11-235L19)	Digoxigenin	Red: MaDig, RaM-TRITC, SwAR-TRITC

Abbreviations: MaDig: Mouse anti-digoxigenin; RaM-TRITC: Rabbit anti-mouse IgG conjugated with TRITC; SwAR-TRITC: Swine anti-rabbit IgG conjugated with TRITC; Bio-GaA: Biotin conjugated goat anti-avidin; Av-FITC: Avidin conjugated with FITC.

Supplemental Table S3:

DNA probe mixtures used for detection of copy number variations for the different chromosomal regions and for detection of viral load using fluorescent in situ hybridization (FISH).

Probe Mix	Number of areas analyzed	Probe mixture/Fluorescent detection		
		Green FITC	Red TRITC	Blue Atto425
1	12	*TERC*	3C	1C
2	12	*TERC*	7C	
3	95	*SOX2*	*SOX17*	
4	62	1C	HPV	

Legend to the Supplemental Figures

Supplemental Figure S1: Transition areas of SOX2 immunostaining patterns in normal squamous epithelium and (adjacent) CIN lesions**.**

a) Transition between SOX2 Pattern 1 and Pattern 2; b) Transition between SOX2 Pattern 1 and Pattern 3; c) Transition between SOX2 Pattern 2 and Pattern 3. The low expression or absence of SOX2 expression in the basal/parabasal cells in Pattern 3 is clearly illustrated in B and C). d) Higher magnification of the upper part of the epithelium showing the brightfield staining of SOX2. Scale bar = 100 μm in a. Magnification in panels a, b, and c is similar.

Supplemental Figure S2: Genetic aberrations in a CIN3 Pattern 3 as detected by FISH. Fluorescence in situ hybridization of a Pattern 3 region in CIN3, targeting copy number variations for the centromere region of chromosome 3 (C3; visualized in red), TERC (visualized in green), combined with DAPI staining for DNA (visualized in blue in a and c) or the centromere region of chromosome 1 (C1; visualized in blue in b and d). The intermediate and superficial cell layers shown in A and B consist of genetically aberrant (aneusomic) cells, while the cells in the basal/parabasal compartments as depicted in C and D show a disomic make up. Scale bar = 25 μm in a. Magnification in panels a, b, c and d is similar.
