# Supplementary figures and images for: SOX2 expression in the pathogenesis of premalignant lesions of the uterine cervix: its histo-topographical distribution distinguishes between low- and high-grade CIN
Source: Histochem Cell Biol. 2022 Aug 9;158(6):545–59. doi: 10.1007/s00418-022-02145-6 (PMC9726813; doi:10.1007/s00418-022-02145-6)

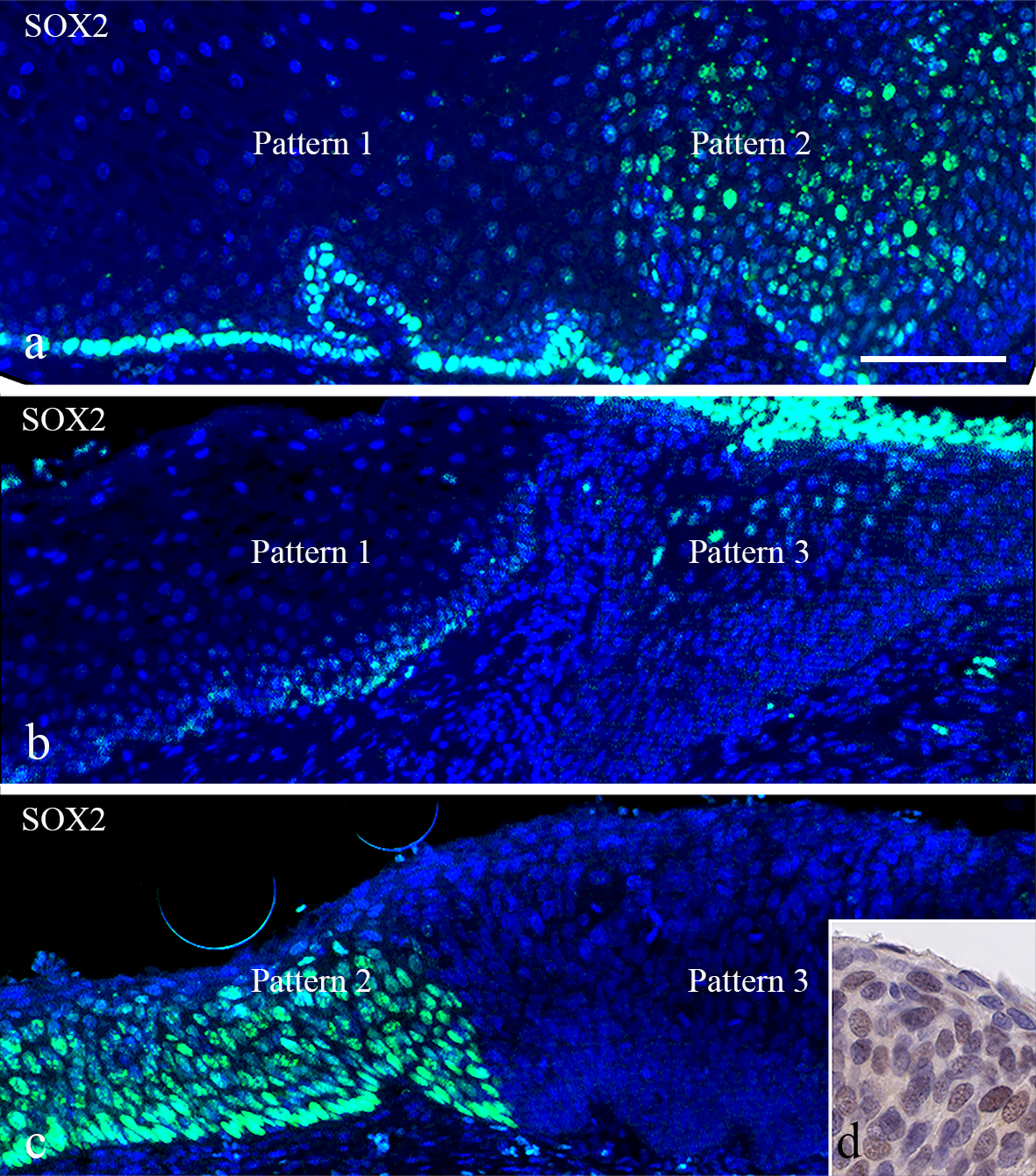

Supplement: Supplementary file 2 — Supplementary file2 (TIF 7512 kb) [file 418_2022_2145_MOESM2_ESM.tif]

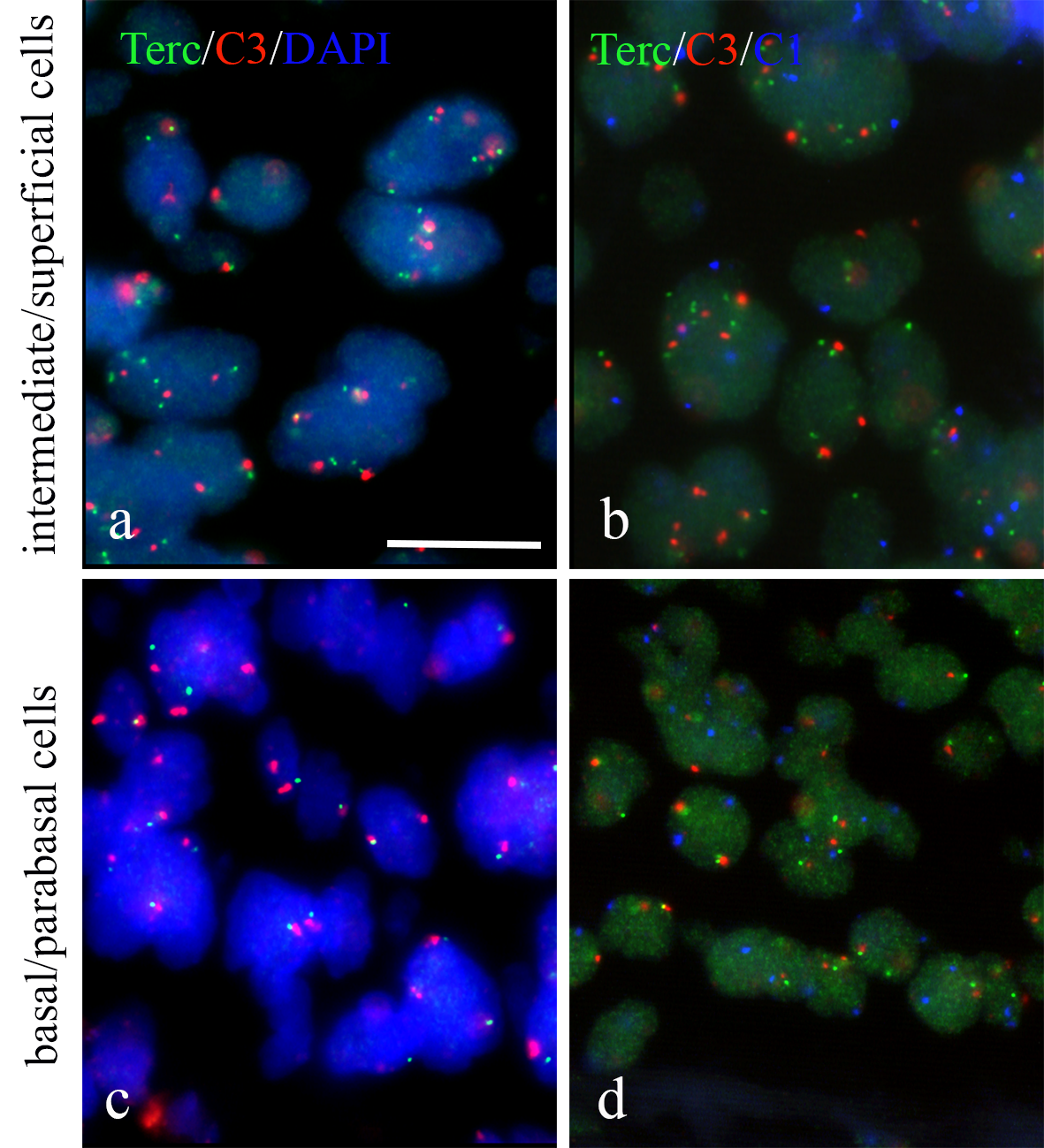

Supplement: Supplementary file 3 — Supplementary file3 (TIF 7285 kb) [file 418_2022_2145_MOESM3_ESM.tif]
